# A Machine Learning Approach to Argo Data Analysis in a Thermocline

**DOI:** 10.3390/s17102225

**Published:** 2017-09-28

**Authors:** Yu Jiang, Yu Gou, Tong Zhang, Kai Wang, Chengquan Hu

**Affiliations:** College of Computer Science and Technology, Jilin University, Changchun 130012, China; gouyu15@mails.jlu.edu.cn (Y.G.); zhangtong15@mails.jlu.edu.cn (T.Z.); kaiwang14@mails.jlu.edu.cn (K.W.)

**Keywords:** thermocline, machine learning, statistical learning, entropy value calculation

## Abstract

With the rapid development of sensor networks, big marine data arises. To efficiently use these data to predict thermoclines, we propose a machine learning approach. We firstly focus on analyzing how temperature, salinity, and geographic location features affect the formation of thermocline. Then, an improved model based on entropy value method for the thermocline selection is demonstrated. The experiments adopt BOA Argo data sets and the experimental results show that our novel model can predict thermoclines and related data effectively.

## 1. Introduction

The marine environment is important to human living and the exploration of the marine environment has become necessary. Thermoclines, an important marine phenomenon, helps a lot in detecting ocean area. The past decades have seen much research on thermoclines, most of which adopt traditional statistical methods to analyze thermoclines. Representative works are as follows. Zhang et al. demonstrated the distribution characteristics of thermoclines and rules of its variety via the mass observation method [1]. They analyzed the ocean by investigating data from 1900 to 2004 of the South China Sea and the sea area around Taiwan, and finally got the distribution of thermoclines in coastal areas in China. Jiang et al. obtained the law of thermoclines’ changes monthly through sampling analysis [2]. Wang also analyzed how the main marine characteristics affected underwater vehicles using the method of mass observation. All methods above commonly used statistical methods when analyzing and processing data.

With the augmentation of modern detection methods, an increased magnitude and complexity of hydrologic data available brings higher capacity of predicting. Our demands for a data processing model are not only for mathematical statistics processing but also for prediction. Statistical methods can obtain the developing trend of the future situation at the premise of the existing situation through analyzing data. The correctness of this trend depends on the stability of the environment. Natural disasters and human activities may bring unpredictable impacts on the open marine environment. In this case, methods based on statistics do not have enough robustness [3].

The underwater environment is tough and it is hard for humans to undertake activities in deep water. Relying on technological ways to perceive the sea is the best solution [4]. In order to better study the thermocline and get access to its properties and application scenarios, we use an underwater sensor network to perceive the marine environment. The sensor network has three functions, which are data acquisition, data processing and data transmission. Underwater sensor networks are composed of stationary or mobile self-organized sensors. At present, the underwater sensor network is applied to environmental monitoring, geographic information acquisition, underwater research, disaster prevention and auxiliary navigation, etc. The first country to carry out underwater sensor networks was the United States. In the 1950s, the United States had deployed acoustic monitoring system—SOSUS in the Atlantic and Pacific oceans. Ocean Network Canada and the University of Victoria deployed the VENUS and NEPTUNE with underwater sensor networks to collect and transmit data. China deployed the first underwater surveillance system of the South China Sea in May 2013.

As the underwater environment is more complicated than expected, current sensor networks may not fully meet the needs. Traditional sensor networks are limited to collect temperature, humidity, position, intensity, pressure, biochemical and other scalar data; however, in reality we need more media information such as video and audio image. Under such demands, a new sensor network—Multimedia Sensor Networks—appears. MSN introduces video, audio and image sensors into traditional sensor networks. The new networks offer us a better perception of the ocean. In addition, multiple platforms’ data fusion offers us access to the marine environment in all situations. We will not only limit to sensor networks. An integrated platform with buoys data, remote sensing data, AUV data and sensor network information helps mankind to know the ocean, protect the ocean and make use of the ocean.

The traditional thermocline selection rule is “the stronger, the better”, i.e., to choose a layer by its definition concerning temperature, depth and strength. In this article, we propose a new thermocline selection method. On the basis of the traditional method, we add more characteristics including the information on geography, salinity, year and month, and then get a new sample criteria-score. In order to ensure thermocline-defining consistency between the traditional selection method and the new method, we set up a one-to-one mapping relationship from strength to score. With mapping strength being critical value to score criteria, we could analyze thermoclines qualitatively and quantitatively. The applicability of this method has been proved by the regional (79.5° S, 79.5° N, 180° W–80° E) experimental results.

**Figure 1:**
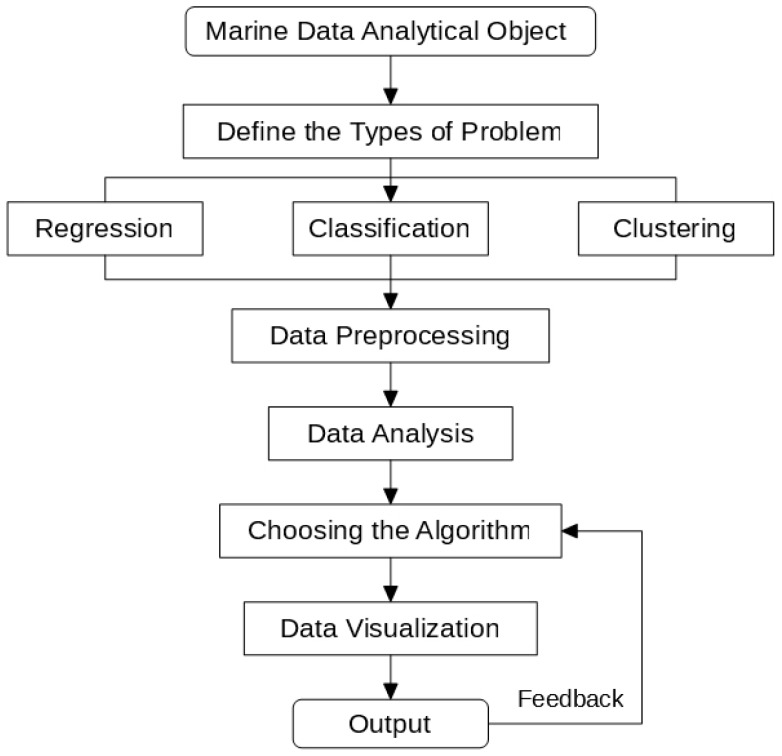
Marine data analytical process.

Besides, we propose a process to tackle marine data-related problems. From Figure 1, we can see that the first step of the research is to define the exact type of the problem, which can be generally divided into regression, classification and clustering. After that, we should preprocess the data into proper forms for analysis. According to the characteristics of the data, we then select an algorithm, which is used for data visualizations. The visualized results then generate feedback to the algorithm model, which improves the model.

The rest of this paper is organized as follows. In Section 2, we perform a brief review of existing research works. In Section 3, we introduce the implementation steps of the methods in detail. In Section 4, experimental evaluation and results are given. Finally, we conclude this work and point out an employment prospect.

## 2. Related Work

In this section, we demonstrate the forming reasons and classification of the thermocline. Through the research development at home and abroad, we here introduce the significance of thermoclines.

### 2.1. Thermocline

A thermocline is a thin but distinct layer in a large body of fluid (e.g., water, such as an ocean or lake; air, such as an atmosphere) in which temperature changes more rapidly with depth than it does in the layers above or below. In the ocean, the thermocline divides the upper mixed layer from the calm deep water below [5]. Salinity is almost stable in the open ocean and pressure only has a slight effect on density, so temperature has become one of the most important factors in density. The temperature of the sea surface is higher than that of the deep ocean; accordingly the sea surface has smaller density compared with deep cold water. Temperature and density in thermoclines change quickly which makes thermoclines an important interface to sea circulation [6].

The reasons for the formation of thermoclines are very complex. In general, temperature is the main factor in water delamination, followed by salinity. For most sea areas, the main power of water delamination comes from intrusion of outland high-density salt water. Water layering and the change of layers’ thickness are closely related to the size and the direction of wind, along with the rainfall strength of the surface layer and low salt water affected by runoff. TIW (Tropical Instability Wave), EUC (Equatorial Under Current) together with ocean currents and atmosphere make seawater come into layers and then form thermoclines. The upper bound of thermoclines mainly mixes inertia gravity wave, Kelvin wave and tropical unstable wave. The sheer weight of TIW and EUC leads to fusion events caused by the lower Richardson number. Lower space appears unstable, which may be ascribed to the absorbing and saturating of waves’ energy. Solar radiation can only maintain the temperature of upper seawater. With the increase of depth, the differences in temperature are getting bigger and bigger. In a certain depth of water, a great temperature gradient is formed, which is called a thermocline. Thus, the cause of thermoclines is very complicated [7,8,9].

### 2.2. Thermocline Classification

Thermocline can be divided into two categories. (1) Seasonal thermoclines are mainly distributed in the mid-latitude area, and most of this kind form and develop in spring and summer. In general, the depth is between 50 and 100 m. The depth and intensity have obvious seasonal changes. In low-latitude areas, the seasonal temperature difference is not big enough to form a layer, so the main thermocline is relatively close to the ocean surface. The depth of the upper bound is roughly between 100 m and 150 m. (2) Main thermoclines (permanent thermoclines) are an important part of the ocean thermal structure, and its strength changes with latitude and longitude. In latitude, the main thermocline near the equator has stronger strength and thinner thickness. The upper bound tends to be shallower. With latitude increased, the main thermocline in the mid-latitude becomes weaker, and the depth of the upper bound gets deeper. The thickness of the thermocline gets a slight increase. At higher latitudes, the main thermocline gets shallow again, and the thickness decreases while the intensity increases. Finally, in the very front area, the thermocline appears in the ocean surface. Along with meridians, the thermoclines around the equator gradually become shallow from west to east.

**Figure 2:**
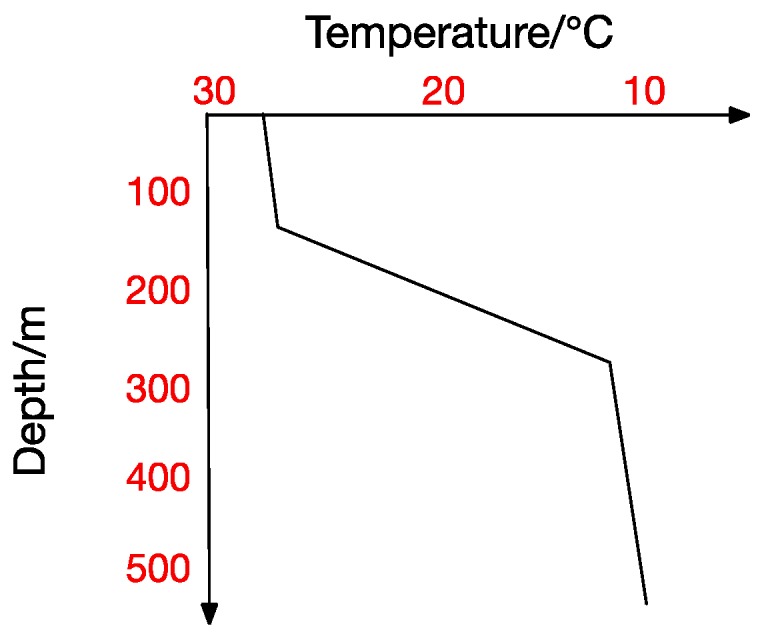
Thermocline.

**Figure 3:**
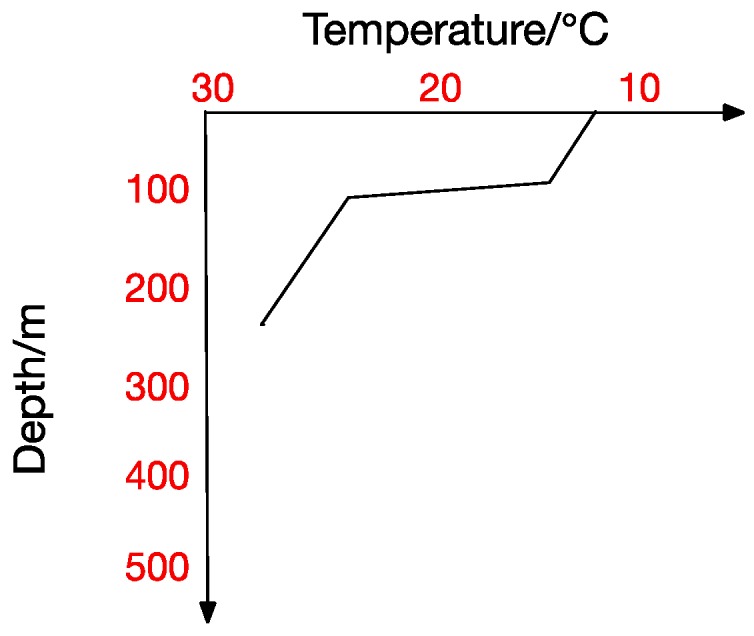
Inverse Thermocline.

**Figure 4:**
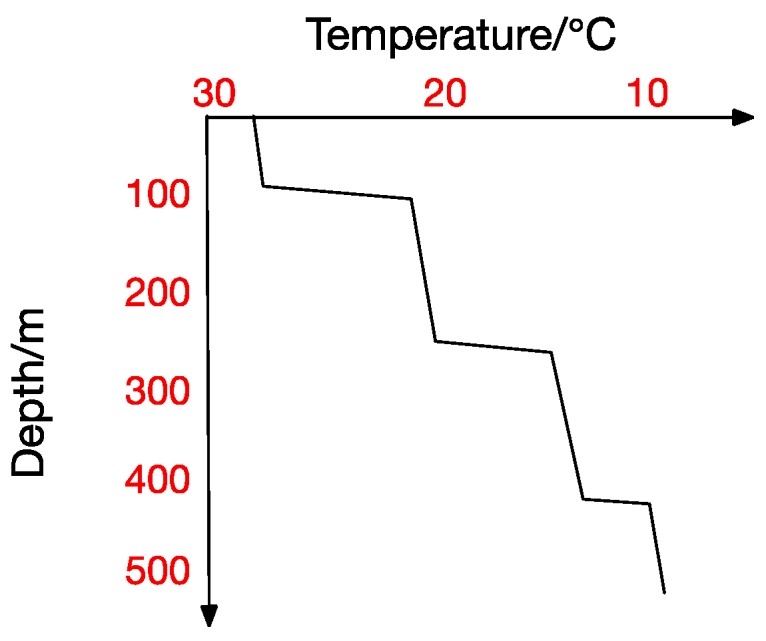
Multi-Thermocline.

**Figure 5:**
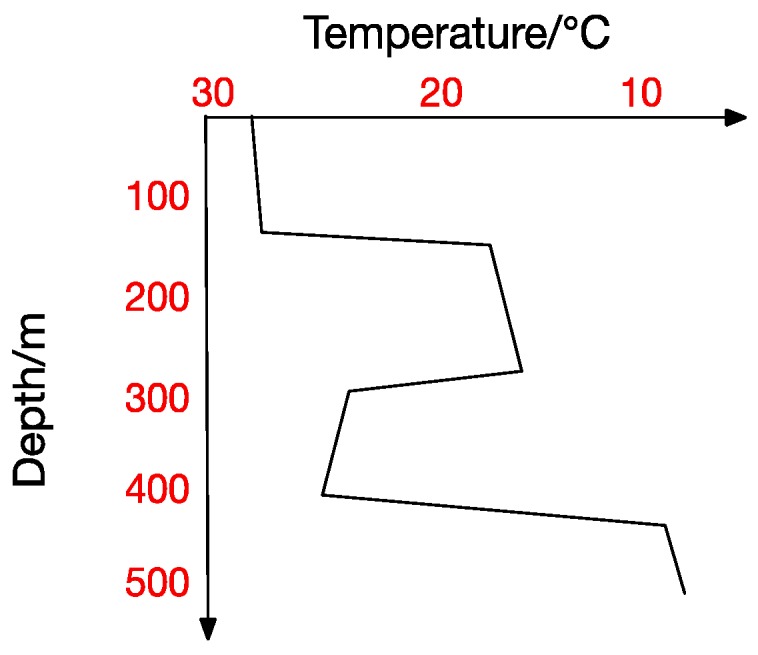
Mix Thermocline.

At the same time, the thermocline can be divided into positive thermocline, reverse thermocline, thermocline and mixed thermocline according to its form. Extracting thermocline kinds and forms from marine data is the foundation for all subsequent studies [10]. Figure 2, Figure 3, Figure 4 and Figure 5 show the four thermocline forms mentioned above respectively.

Figure 4 and Figure 5 also point out that ocean temperature changes with depth in a complicated way, which makes a temperature-changing prediction model a tougher task. The result analyzed in traditional statistical methods is actually a trend representing the correlation between values. In contrast, machine-learning methods usually get the inner correlation among water features, which is obviously closer dependency. Hydrological characteristics may become abnormal under certain conditions, which will lead to a deviation from previous predicting trends and thus form a relatively big error. Machine learning methods seem more suitable for this situation. In a machine learning method, we can get the interior correlation, and values may change while correlation is stable. According to the correlation, predicted values change with characteristic values and then get a prediction model closer to the reality.

### 2.3. Significance of Thermocline Research

Thermocline research is of great significance not only in the academic field but also the production, living and military field. In the military field, for example, sound waves in seawater are affected by the longitudinal and vertical section in a large degree, so it is vital for submarine warfare and anti-submarine warfare to figure out how thermoclines are influenced by temperature and density, and how thermoclines influences sound propagation. Thermoclines are also known as the liquid seabed. Around the bottom of the sea exists an invisible layer, which makes the sound waves’ spread reflect. The sound waves ripping into the liquid seabed can also produce refraction. Therefore, in the bottom of the liquid seabed, a hidden submarine can avoid enemies’ sonar effectively. Only by figuring out the changing rules of the sea temperature can we take advantage of this factor better in the future.

### 2.4. Thermocline Research Actuality

In the past, researchers often used sensor nodes and buoys to collect information of hydrological characteristics, such as temperature, density, pressure and oxygen underwater. The information at first was transferred to the ground station through cable and then sent to the server. In recent years, researchers started to collect data using AUVs, which can collect data dynamically. There are several famous thermocline-researching institutions, such as MIT Research institutions, the Woods Hole Oceanographic Institute (http://www.whoi.edu/), the Monterey Bay Aquarium Research Institute (http://www.mbari.org/), Russia’s Far East Ocean Research Institute and the University of Porto. The University of Porto used simplified models of thermocline with the vertical gradient method. The vertical gradient method is a common observation analysis method for thermoclines. Research institutions such as MIT vertically analyzed the thermocline data (https://auvlab.mit.edu/) collected with the vertical gradient method. Then, they integrated time and space, and put forward the “Adaptive Environmental Assessment” (Autonomous the Adaptive Environmental Assessment, AAEA) algorithm, in the case of updating AUVs’ navigation information in an unsupervised judgment area [11].

The use of Argo data for marine research has become a hot spot internationally. The heat budget in the upper ocean (0–300 m) of the North Atlantic from 20–60° N is studied using data from Argo profiling floats from 1999 to 2005 and the NCEP/NCAR and NOC surface flux datasets [12]. It is investigated that the Argo profiling float dataset can be utilized to estimate the temperature and heat storage of the upper ocean in the North Atlantic [13]. In situ observations from the autonomous Argo float array are used to assess the heat content change of the basin-averaged ocean driven by tropical cyclones (TCs) in the North Pacific for 2000–2008 [14]. The means, variances, and three-dimensional spatial covariances of the ocean temperature and salinity anomalies in the upper 1400 m layer have been estimated using data of the Argo profiling floats from 2005 to 2007 [15]. It is demonstrated how to use unsupervised classification of the Argo temperature profiles to achieve coherent thermal patterns [16].

In recent years, Chinese researchers from Harbin Engineering University, Shanghai Jiao Tong University, Shenyang Institute of Automation, Chinese Academy of Sciences and the Chinese Academy of Sciences Institute of Marine and so on mainly aimed at thermocline characteristics of Chinese sea areas. Professor Ge used the step function approximation method to calculate the shelf sea thermocline characteristics—step function, research standard thermocline and inverse thermocline—which can fit the temperature curve perfectly [10]. Zhang from the Navy Marine Hydro-Meteorological Center compared the non-equidistant differential method, the vertical gradient method and a 7.2x smoothing algorithm in ocean thermocline eigenvalue analysis and calculation [17]. He aimed at excluding and merging multi-thermoclines as long as thick ones in complicated waters. Zhang from the Dalian Naval Academy expounded the vertical gradient method and optimal segmentation. He compared and analyzed the performance of these two approaches when determining a thermocline boundary [18]. Thermocline changes from season to season. From winter to fall, these are non-leap period, growth period, strong period and fade period. This means time will also be an important factor in our study. Existing research works are basically according to quarter or month. Given that our research mainly depends on thermoclines, we should try to refine time to form the most accurate thermocline-changing map. At the same time, due to the large number of data, data analysis and processing become the first step in our study. Common methods to interpolate data are as follows: inverse distance weighted interpolation, the Kriging interpolation method, the minimum curvature method, the multiple regression method, the natural adjacent points interpolation method, the nearest neighbor interpolation method, local polynomial method and so on. In this paper, we use the k nearest neighbor interpolation method to deal with the data. After that we will classify the collected data through the machine learning method. After analyzing data, we need to give each group of data a tag, which represents whether the point is in the thermocline. Then we obtain thermocline distribution by calculation and statistical analysis. Further research in the study area aims at setting correlation among time, temperature and depth and acquiring thermocline distribution. With all the information obtained above, we can finally draw a 3D map of temperature, depth and intensity. With the research done thoroughly, the value of the thermocline will become larger than we expect.

## 3. Methodology

### 3.1. Determine the Thermocline

To study the thermocline, we need to judge whether the thermocline exists in the area. Then we will determine the upper bound and lower bound of the thermocline, and find out the exact location of the thermocline. There are many calculating methods to determine upper and lower bounds for the thermocline. The common ones are the S-T method, vertical gradient method and maximum curvature point method [19]. The so-called depth of the upper bound of the thermocline is the depth from surface to the thermocline intensity threshold. We use the maximum curvature point method and vertical gradient method together to calculate the upper thermocline bound. Here, we briefly explain the concept of thermocline strength, which refers to the quotient of temperature difference divided by depth difference.

The current thermocline strength evaluation standard follows the Standard of Marine Survey, in which shallow water depth (less than 200 m) intensity threshold is 0.2 °C/m and deep depth (more than 200 m) intensity threshold is 0.05 °C/m [20]. As the temperature of the study area changes little after 200~300 m, called a static area, we use 0.2 °C/m as the critical value of judging a thermocline for unified calculation so that we can filter out a thermocline of better quality.

We can know the maximum regional strength through the vertical gradient method and the maximum curvature point method. If the maximum value cannot reach the critical value, no thermocline exists and the position can be excluded from consideration, which can reduce the sample space to some extent. It is not advisable to determine the upper bound by a single method, and only after being integrated with multiple methods can the best result be found. We here present how to determine a thermocline. First of all, we need to input the matrix of ocean temperature and depth. Combining the vertical gradient method with the maximum curvature point method, we use this matrix to calculate each point’s temperature strength according to the definition. With the calculated strength, we then screen out those points meeting the standard of thermocline (the temperature is more than 0.2 °C/m). If the point is in the thermocline, we write the data into a specific file or the data is discarded.

After determining the thermocline, we tag all samples. For the convenience of the following work, we will introduce all the characteristics used in this paper and all the samples having these characteristics. Table 1 is a table of characteristics and their brief descriptions.

### 3.2. Merge the Thermocline

There usually exist 3–5 thermoclines at the same position vertically. These thermoclines need to be merged. It is a fact that thin thermoclines cannot meet the needs of underwater vehicles or buoyancy’s diving movement range solely, and a bigger thickness means a higher temperature span. It is good to see more temperature changes, as it is momentous for our research. Note that instruments and some hydrological characteristics will introduce disturbances to data we use. The fluctuations can produce many thinner thermoclines [17]. Their thickness is commonly between 1–2 m. Such thermoclines have all the features of a thermocline such as large span, small thickness, big strength and so on. Although the features are obvious, such layers cannot be viewed as real thermoclines. In order to eliminate fluctuations in our experiments, we decided to interpolate data vertically to a 1 m-interval data, and filter out the thermoclines whose thickness are less than 5 m. The process of merging thermoclines is as follows. After determining a thermocline, we get several untreated thermoclines, and we need to separate them according to their thicknesses. Those of thickness greater than 5 m are needed. If the nearby thermoclines have an interval between current lower bound and next upper bound more than 5 m, the current thermocline is set as an independent layer; if not they are merged with the adjacent thermoclines. After this, the thermoclines produced by fluctuations are filtered out.

### 3.3. Choose the Thermocline

In traditional ocean field, thermocline-related problems are always computed with statistical knowledge. In previous studies, thermocline selection follows the principle of “the stronger, the better”, i.e., the thermocline with the maximum intensity value is optimal at a single vertical gradient [17]. If the intensity of two adjacent thermoclines is close and thickness difference is bigger, the larger thickness one is selected. If the intensity and thicknesses are all the same, the one whose strength is slightly larger is chosen.

It is not hard to see that traditional statistical methods sort several thermocline characteristics by importance. However, this approach is only a rough one. When considering strength, other hydrological characteristics may be neglected. Hence, in this paper we are trying to use a machine learning method—“entropy value method”—to analyze and compare several characteristics, and sort them by their importance. Thermocline has a lot of characteristics such as strength, depth, salinity, etc. However, they are not equally important towards thermocline. Our purpose is to comprehensively consider these factors in each sample to get its quality. The advantage is that we can comprehensively evaluate each sample and each attribute, reduce the experimental error caused by statistical error and select a better thermocline.

While using “entropy value method” to calculate, the following concepts are needed. Here is a brief introduction.

*Information entropy*: an indicator to measure sample collection’s purity. Assume that the proportion of the first k classes in the data set D sample is $p_{k}$ (k = 1, 2, 3, …, |y|)

$$Ent(D)=\sum_{k=1}^{\left|y\right|}p_{k}\log_{2}^{p_{k}}$$

The more uncertain of the variables, the greater the entropy values are.

*Information gain*: The bigger the information gain, the greater the purity gained by using attributes “a” to divide.

The steps of “entropy value method” are shown in Algorithm 1.

**Algorithm 1 Entropy Value Method***STEP 1*. *Select n samples attributes, then $x_{ij}$ shall be the value of the i-th sample’s the j-th attribute (i =* 1, 2*, …, n; j =* 1*, 2, …, m).* *STEP 2*. *Normalize the index and make the homogeneity data a homogeneity.* Make $$x_{ij}=|x_{ij}|$$
$$x_{ij}=\frac{x_{ij}-\min\left\{x_{ij},\cdots ,x_{nj}\right\}}{\max\left\{x_{1j},jx_{nj}\right\}-\min\left\{x_{ij},\cdots ,x_{nj}\right\}}$$
*then $x_{ij}$ shall be the value of the i-th sample’s j-th attribute (i =* 1*,* 2*, …*, *n; j =* 1*,* 2*, …, m).* *STEP 3*. *Calculate the proportion of the i-th sample in j-th attribute.*
$$p_{ij}=\frac{x_{ij}}{\sum_{i=1}^{n}x_{ij}}\;(i\;=1,\;\ldots ,\;n;\;j\;=1,\;\ldots ,\;m)$$
*STEP 4*. *Calculate the entropy value of the j-th attribute.*
$$e_{j}=-k\sum_{i=1}^{n}p_{ij}\ln(p_{ij})$$
$$k=\frac{1}{\ln(n)}>0,\;e_{j}\geq 0$$
*STEP 5*. *Calculate the information gain*. $d_{j}=1-e_{j}$
*STEP 6*. *Calculate the weights of each index.*
$$w_{j}=\frac{d_{j}}{\sum_{j=1}^{m}d_{j}}$$
*STEP 7*. *Calculate the comprehensive score of each sample.* $s_{i}=\sum_{j=1}^{m}w_{j}p_{ij}$ where, ***s*** is the comprehensive score of each sample when it comes to form a thermocline. ***w*** is the important degree of each attribute for the formation of thermocline.

Compared with the traditional principle “the stronger, the better”, we propose a new method to evaluate thermoclines. Each point that consists in a thermocline has its own composite score “s”. We add all these “s” values and then calculate the average for each thermocline. Compared with the traditional principle “the stronger, the better”, the machine learning method of “entropy value method” combines all the attribute values. Apparently, such method is more comprehensive, and more accurate. The “entropy value method” can remedy the defects caused by only considering strength without other factors, and make the result more objective.

### 3.4. Use Machine Leaning Method to Predict

Collecting historical data is to summarize rules and get the predictions for future situations. These goals need us to use machine learning methods. Machine learning is an inevitable outcome with the development of artificial intelligence. As the core of artificial intelligence, constructing machine learning frameworks has the following steps. (1) Divide data into training set and testing set. (2) Use the training set and its characteristic vectors to train algorithm. (3) Use the testing set to evaluate algorithm learnt earlier [4]. This paper involves the following terms of machine learning.

*Data set*: A batch of data collected about the object. In this paper, the data sets are from the global ocean Argo grid data set (BOA Argo), recording the temperature from 2012~2015 about the hydrology factors’ changing such as temperature and salinity.

*Sample*: A record in the data set. A sample is a description of an event or an object, also known as a characteristic vector, such as (latitude = 16.5°, longitude = 115.5°, year = 2015, month = 7, depth = 97 m, temperature = 29.334381 °C, temperature strength = 0.295814, salinity = 33.688723).

*Characteristics*: Reflect event or object’s performance and character, such as “depth”, “strength” “temperature”, etc.

*Attribute value*: Values of attributes.

**Figure 6:**
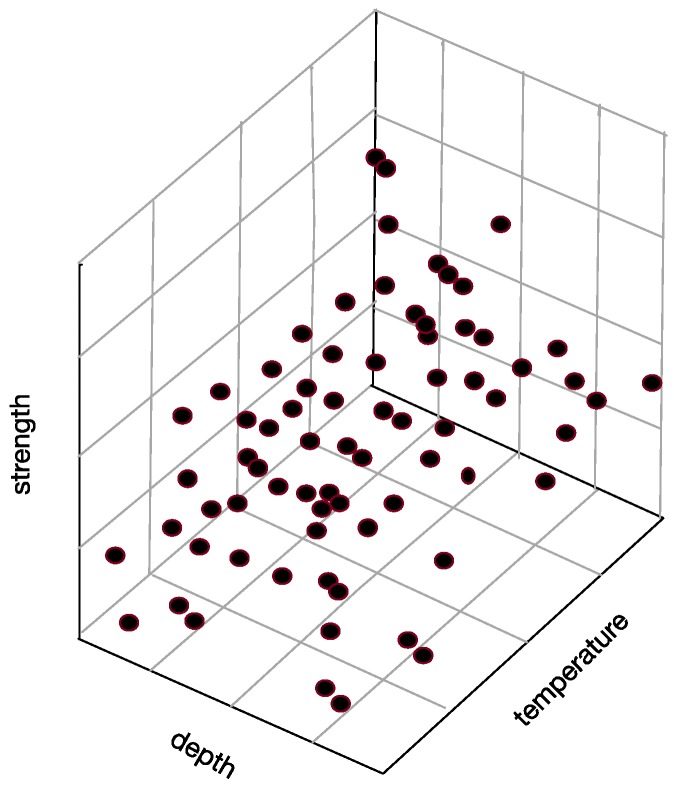
Three-dimensional state space describing underwater thermocline.

*Sample space*: The space consists of attributes. For example, set “strength”, “depth” and “temperature” as three axes in Figure 6, and then they form a three-dimensional state space describing underwater thermocline.

Every sample in thermocline can find its corresponding coordinates in the sample space, which means that each point in the sample space corresponds to a sample.

*Training set*: Data set used to train model.

*Testing set*: Data set used to test learning model’s performance. 

According to the features of the predictive content, machine learning tasks can be divided into the following categories. (1) Classification: predict the discrete value. (2) Regression: predict the continuous value. The main task of this paper is to classify the data and separate the thermocline area from other no jump areas.

The Argo data are unmarked and our task is to mark these data according to whether they are in the thermocline. The water data collected are the basis of our rule summary work and future predictions. That is to say, we are going to use this large data set for both training and testing at the same time. Because the sample’s data structure is relatively simple in our research, we will divide the data set into training set S and testing set T through “set aside method”. At first, we separate these data into two parts according to whether their strength reaches the critical temperature strength value. In addition, then we take 3/5 as the training set, 2/5 as a testing set from positive cases and negative cases respectively. This ensures the consistency of positive and negative cases’ distribution [4].

When forming the state space, we obtain the rule model containing local changing rules at the same time. Where there is a new data set, there is an instant result of the thermoclines’ distribution. This will prevent us from meaningless and time-consuming work.

### 3.5. Map Thermocline Strength to Score

From the definition of a thermocline, we use temperature and depth to calculate each vertical position’s strength. Through the entropy value method, we get each sample’s scores comprehensively considering all the relative factors. As mentioned before, the score contains more information than strength. At first, we need to make sure that the two methods can select the same samples, namely the thermocline. Then we will map the strength to score. The thermocline is quantitatively analyzed on the premise of same select criteria. The thermocline with the higher score will be chosen.

## 4. Experimental Evaluation

### 4.1. Data Sets

The data used in this paper is from the 2012~2015 global Marine Argo grid data set (BOA Argo). The chosen data is from the China Argo real-time data center (http://www.argo.org.cn/) from January 2004 to December 2015 global ocean (79.5° S–79.5° N, 180° W–180° E) Argo’s temperature and salinity. These data sets have passed profile data real-time and part-time delay quality control in all countries. The data is recorded monthly, and horizontal resolution is $1^{\circ }\times 1^{\circ }$. From sea level to 2000 m beneath water, the sea is divided into 26 layers vertically. This production is from the State Oceanic Administration of Marine Environment Key Laboratory of Information Security Technology and National Marine Information Center. On this basis, we reprocess the data using the k-nearest interpolation method vertically. As a result, we make the data set a 1 m-interval data product. Our studies focus on the thermocline; however, a large number of visualization experiments have shown that seawater beneath 500 m entered a static state and no longer produce thermoclines. Hence, the area under 500 m is meaningless to our study. When reprocessing data, we divide the sea into 500 layers with a 1 m-interval from sea level to 500 m underwater. Our present study is in its infancy, and we will deal with the data to in the further study, which will be released on the website.

**Figure 7:**
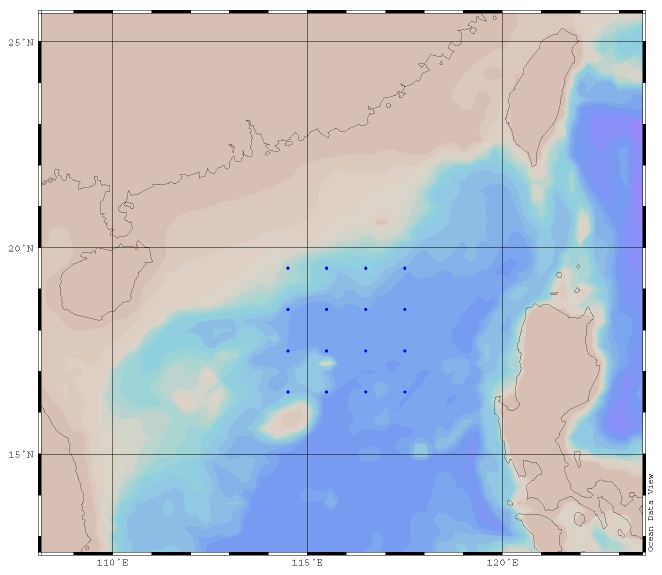
The samples distribution map.

Figure 7 describes the distribution map of 100,000 samples selected in BOA Argo. Table 2 is the result of the accuracy analysis of 100,000 samples selected in BOA Argo. It is demonstrated that the accuracy increases with the increase of the training set samples, which is in accordance with the characteristics of the accuracy and training set in the machine learning.

### 4.2. Determine the Thermocline

We start from the definition of temperature strength to determine the thermocline. Make the temperature *t*, depth *d*, layer number *n*, and temperature strength of *l*.

$$l=\frac{t_{n+1}-t_{n-1}}{d_{n+1}-d_{n-1}}\;\;n=2,3,4,\cdots$$

There exists no thermocline in the surface, and the ignorance of the first layer will not influence our test. From the previous thermocline in figures (Figure 2, Figure 3, Figure 4 and Figure 5), we can observe that there may exist thermocline and reverse thermocline at the same time, which means temperature strength may be positive or negative. To simplify calculation, here we regulate

l=|l|>0

According to the judging standard of temperature strength, we filter the “*l* > 0.2” samples and write them into data.txt file. The file will be used when merging and selecting thermoclines.

**Figure 8:**
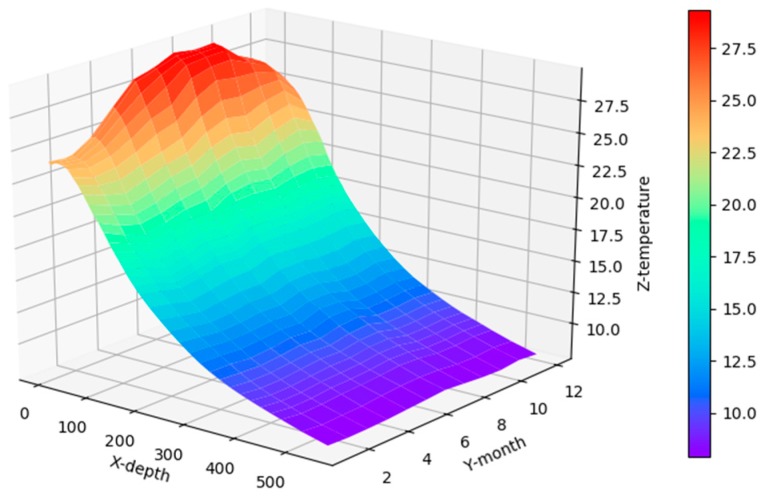
Three-dimensional Correlation among temperature, depth and time.

**Figure 9:**
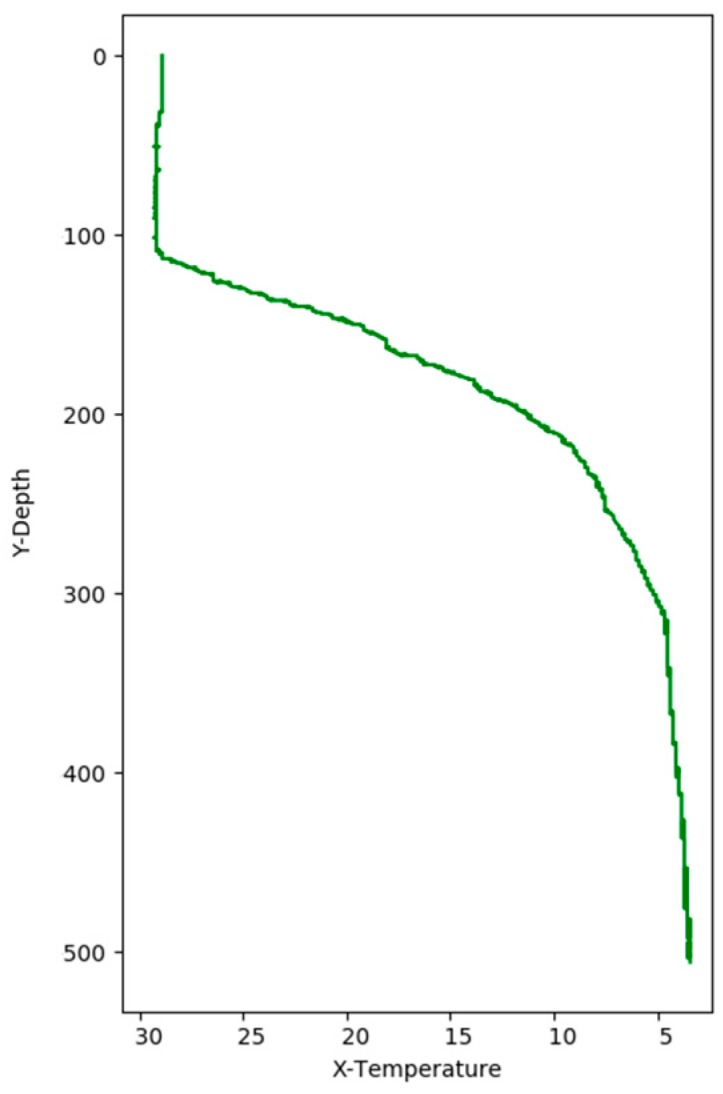
Correlation between depth and temperature.

**Figure 10:**
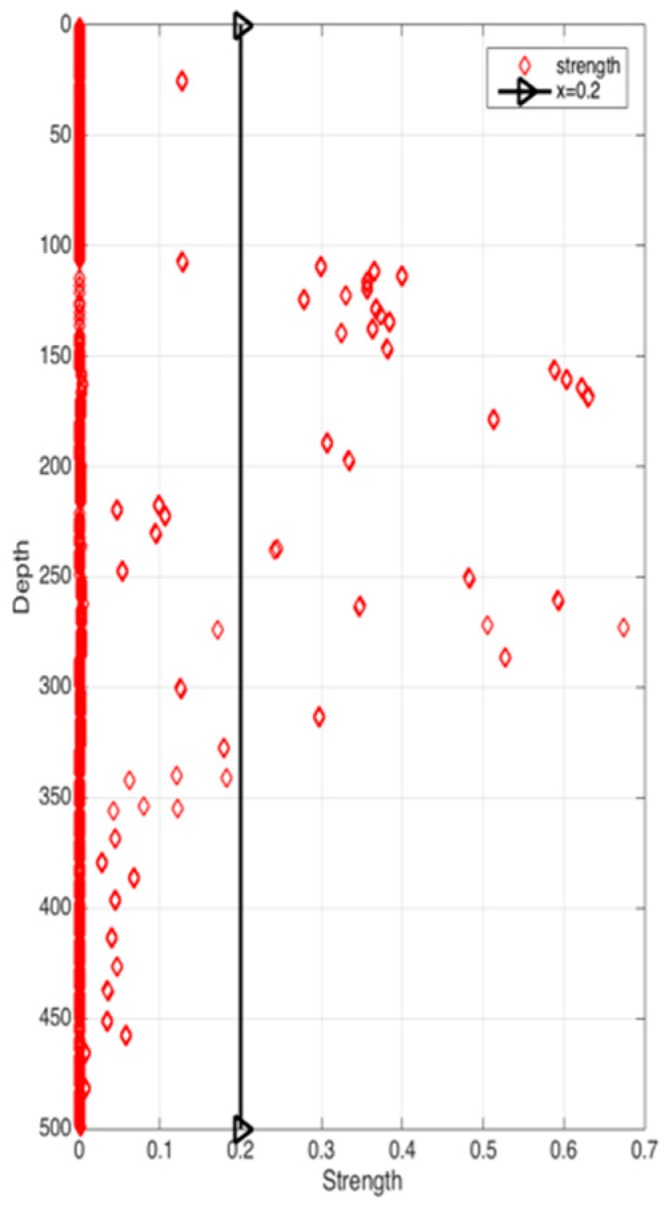
Correlation between depth and strength.

**Figure 11:**
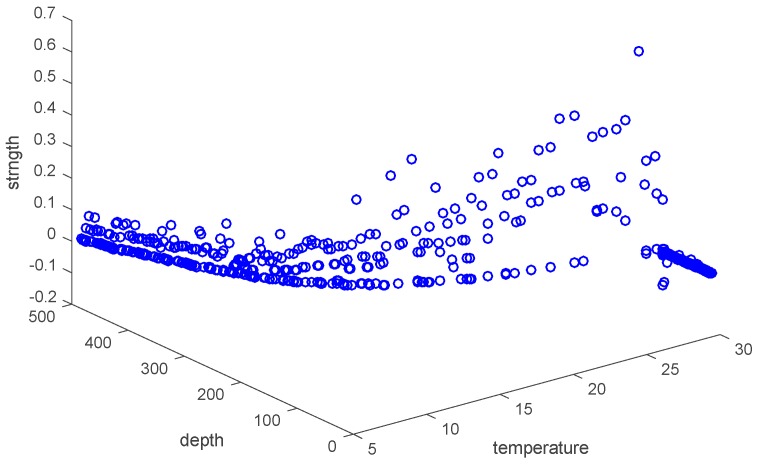
Attribute space made up of temperature, strength and depth.

Figure 8 shows the correlation among temperature, depth and month. Red represents higher temperature while blue stands for lower temperature. From Figure 9 we can find that temperature is changing with depth. From surface to 100 m underwater, the temperature stays stable. Between 100 m and 300 m, temperature falls sharply. Within the depth of 200 m or so there are more than 20 °C temperature difference. After 300 m in depth, the water gets into a stable state again and temperature changes little. Points in Figure 10 illustrate the correlation between depth and strength. Diamond is on behalf of a certain depth’s thermocline strength. x = 0.2 in the black line represents the thermocline intensity threshold. The points on the left side of the line are not in the thermocline area, while those points on the right side are in the interior of a thermocline. Similar to Figure 10’s observation, points satisfied with the critical strength are more likely at 100 m to 300 m underwater. Figure 11 is the attribute space made up of temperature, strength and depth. Each hollow circle represents a sample.

### 4.3. Choose the Thermocline

Merge the samples, whose depth differences are within 5 m, to one thermocline and get a certain number of thermoclines. Then we will take the “entropy value method” to choose among these merged thermoclines.

We use MATLAB_R2015b as the experiment platform and finally get the result.

**Figure 12:**
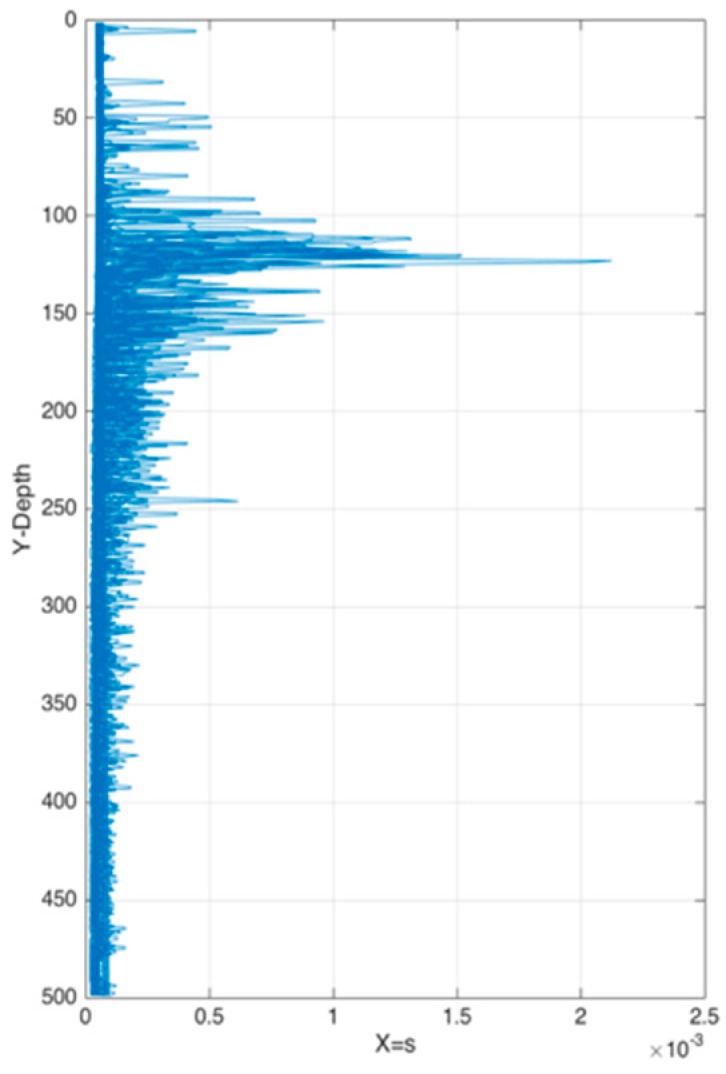
Weights s of each point from sea surface to 500 m underwater.

Table 3 shows the weights changing of each attribute when forming the thermocline in the experimental area monthly. Figure 12 represents weights *s* of each point from sea surface to 500 m underwater. As we mentioned earlier in this paper, temperature strength is the most important factor when forming a thermocline. Temperature gradient is consisted of temperature and depth. The gradient is influenced more by temperature than depth. As another hydrological characteristic, salinity also affects the formation of the thermocline slightly. Although from the previous experiments we can conclude that the thermoclines are distributed in the region of 100–300 m in depth, Figure 12 shows good-quality samples distributed between 100 m and 150 m in depth. It is worth noting that the longitude and latitude occupy a considerable proportion in the formation of the thermocline. This conclusion perfectly proved that the “the stronger, the better” principle is not that rigorous and machine learning can make an improvement.

### 4.4. Mapping from Strength to Score

Initially, it is necessary to ensure whether the two methods can select the same samples, namely thermocline. We use Matlab_R2015b as the experimental platform and finally got the results.

**Figure 13:**
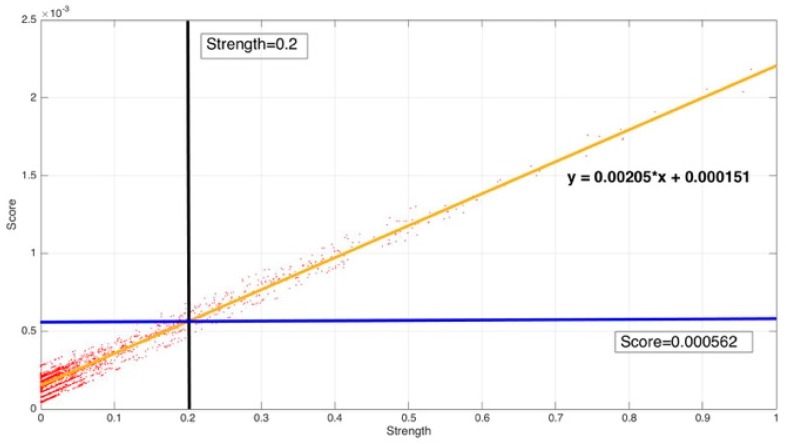
Correlation between strength and score.

From Figure 13, it can be found that the map relation between strength and score is fitted as: y=0.00205x+0.000151.

The strength criteria and the corresponding score criteria select the same samples. The result satisfied our assumption.

### 4.5. Marine Data Curve Fitting

In this step we still use MATLAB_R2015b to fit the ocean data curve.

**Figure 14:**
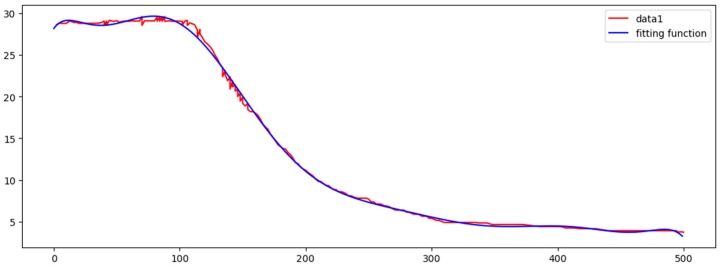
Marine data curve and the fitting function curve.

**Figure 15:**
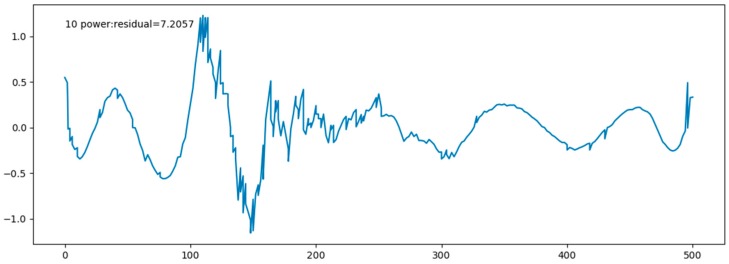
Residual plot.

Figure 14 shows marine data curve and the fitting function curve, which are fitting perfectly. Figure 15 shows the residual plot. In the table, fitting function and all the residuals are given.

After several fitting attempts, we found that a 10-times function can fit the data better. Table 4 shows fitting function and all the residuals. It is important that an overfitting phenomenon may appear in a higher-order fitting, so we will try various ways to reduce overfitting phenomenon in the following works.

## 5. Conclusions and Future Work

The traditional thermocline selection rule is “the stronger, the better”, i.e., choose a layer by its definition concerning temperature, depth and strength. In this article, we proposed a new thermocline-choosing method. On the basis of the traditional method, we add more characteristics including geography information, year/month information and salinity, and get a new sample criteria-score. In order to ensure thermocline-defining consistency between traditional selection method and the new method, we set up a one-to-one mapping relation from strength to score. With mapping strength being of critical value to score criteria, we can qualitatively and quantitatively analyze and the thermocline. The applicability of this method has been proved by the regional (79.5° S, 79.5° N, 180° W–180° E) experimental results.

In the future, we will use hydrological data sets to make a decision tree. The “entropy value method” mentioned in this paper is on the basis of the information gain to divide characteristics aimed at each characteristic’s weight and each sample’s comprehensive score. The key to making a decision tree is the division of characteristics. ID3 decision tree learning algorithms based on information gain, C4.5 decision tree algorithms based on information gain ratio and CART decision trees based on Gini coefficients are the three commonly used divide methods. However, the ID3 algorithm is inclined toward characteristics with more dereferences, the C4.5 algorithm prefers those having fewer dereferences, and the CART algorithm is weak in continuous value prediction. Comparing all these methods, we should try to combine their advantages and balance their drawbacks. In terms of the decision tree, there always exists an “overfitting” phenomenon in its learning procedure. To lower the risk caused by overfitting, we plan to use pruning or the random forest method. These two methods also need to be analyzed and measured. The detailed steps about how to choose an appropriate method are of great significance.

## Figures and Tables

**Table 1:** Characteristics and their descriptions.

Characteristics	Brief Descriptions
Latitude/Longitude	Where the data were collected
Year/Month	When the data were collected
Depth	Distance from sea surface to the point
Temperature	Temperature of the point
Strength	Temperature difference/depth difference
Salinity	Hydrological characteristics

**Table 2:** Accuracy vs size of training data.

Train Set	Test Set	Accuracy
5000	95,000	0.99245
10,000	90,000	0.99486
15,000	85,000	0.99570
20,000	80,000	0.99623
25,000	75,000	0.99671
30,000	70,000	0.99718
40,000	60,000	0.99741
50,000	50,000	0.99763
70,000	30,000	0.99801

**Table 3:** Weights of each attribute when forming thermocline monthly.

w	Latitude	Longitude	Depth	Temperature	Strength	Salinity
January	0.1266	0.1266	0.0752	0.1737	0.4867	0.0112
February	0.1360	0.1360	0.0808	0.1810	0.4554	0.0108
March	0.1391	0.1391	0.0826	0.1827	0.4454	0.0112
April	0.1388	0.1388	0.0825	0.1867	0.4388	0.0144
May	0.1361	0.1361	0.0809	0.1885	0.4365	0.0218
June	0.1349	0.1349	0.0802	0.1866	0.4399	0.0235
July	0.1350	0.1350	0.0802	0.1905	0.4500	0.0094
August	0.1338	0.1338	0.0795	0.1926	0.4490	0.0112
September	0.1339	0.1339	0.0796	0.1910	0.4534	0.0082
October	0.1339	0.1339	0.0796	0.1898	0.4519	0.0110
November	0.1337	0.1337	0.0795	0.1909	0.4553	0.0069
December	0.1342	0.1342	0.0797	0.1908	0.4544	0.0067

**Table 4:** Fitting function and all the residuals.

Fitting Function	y = p1 × z^10^ + p2 × z^9^ + p3 × z^8^ + p4 × z^7^ + p5 × z^6^ + p6 × z^5^ + p7 × z^4^ + p8 × z^3^ + p9 × z^2^ + p10 × z + p11	
Coefficient	P1	−1.1173
	P2	0.75808
	P3	8.3512
	P4	−6.2657
	P5	−20.714
	P6	17.92
	P7	15.847
	P8	−18.203
	P9	6.6455
	P10	−6.0711
	P11	7.3533
Residual	7.2057	
z	(x-mu)/sigma	
mu	249.5	
sigma	144.48	

## References

[1] Zhang M., Liu J., Mao K., Li Y., Zhang X., Shi Y. (2006). The general distribution characteristics of thermocline of China Sea. Mar. Forecast..

[2] Jiang G., Hao S., Tao D. (2011). Monthly analysis of characteristics of northern South China Sea thermocline change. Mar. Forecast..

[3] Wang K. (2015). Influence of main ocean environments on the navigation of underwater vehicles. CAAI Trans. Intell. Syst..

[4] Zhou Z. (2016). Machine Learning.

[5] Hosoda S., Minato S., Shikama N. (2006). Seasonal temperature variation below the thermocline detected by Argo floats. Geophys. Res. Lett..

[6] Yin J., Wang Y., Xu J. (2006). Daya bay thermocline formation and its influence to the related environmental elements. Mar. Sci. Bull..

[7] Chao J., Gao X., Feng L. (2007). Shear instability of basic flow in a two-layer equatorial ocean model. Acta Meteorol. Sin..

[8] Chen X., Hu B., Li W. (2009). The equator unstable wave numerical simulation analysis of the impact of air-sea interaction. Chin. J. Atmos. Sci..

[9] Liu C., Köhl A., Liu Z., Wang F., Stammer D. (2016). Deep-reaching thermocline mixing in the equatorial pacific cold tongue. Nat. Commun..

[10] Ge R., Qiao F., Yu F. (2003). A method of calculating the shelf sea thermocline characteristics-step function approximation method. Adv. Mar. Sci..

[11] Liu J., Li D., Ji D. (2014). The methods of detecting thermoclines in oceans by AUV: A review. J. Ocean Technol..

[12] Wells N.C., Josey S.A., Hadfield R.E. (2009). Towards closure of regional heat budgets in the North Atlantic using Argo floats and surface flux datasets. Ocean Sci..

[13] Hadfield R.E., Wells N.C., Josey S.A., Hirschi J.J.-M. (2007). On the accuracy of North Atlantic temperature and heat storage fields from Argo. J. Geophys. Res. Oceans.

[14] Park J.J., Kwon Y., Price J.F. (2011). Argo array observation of ocean heat content changes induced by tropical cyclones in the north Pacific. J. Geophys. Res. Atmos..

[15] Resnyanskii Y.D., Tsyrulnikov M.D., Strukov B.S., Zelenko A.A. (2010). Statistical structure of spatial variability of the ocean thermohaline fields from argo profiling data, 2005–2007. Oceanology.

[16] Maze G., Mercier H., Fablet R., Tandeo P., Radcenco M.L., Lenca P., Feuche C., Le Goff C. (2017). Coherent heat patterns revealed by unsupervised classification of Argo temperature profiles in the North Atlantic Ocean. Prog. Oceanogr..

[17] Zhang X., Zhang X., Li Y. (2011). Ocean thermocline eigenvalue analysis and calculation. Mar. Forecast..

[18] Zhang X., Zhang Y., Nie B. (2008). Comparative analysis of thermocline boundary between vertical gradient method and optimal segmentation method. Mar. Sci. Bull..

[19] Jiang B., Wu X., Ding J. (2016). Comparison of the calculation methods of the thermocline depth of the South China Sea. Mar. Sci. Bull..

[20] Hao J., Chen Y., Wang F., Lin P. (2012). Seasonal thermocline in the China Seas and northwestern Pacific Ocean. J. Geophys. Res. Oceans.

